# Content and Nutritional Evaluation of Zinc in PDO and Traditional Italian Cheeses

**DOI:** 10.3390/molecules26206300

**Published:** 2021-10-18

**Authors:** Pamela Manzi, Maria Gabriella Di Costanzo, Mena Ritota

**Affiliations:** Consiglio per la Ricerca in Agricoltura e L’analisi Dell’economia Agraria, Centro di Ricerca Alimenti e Nutrizione, Via Ardeatina 546, 00178 Rome, Italy; mariagabriella.dicostanzo@crea.gov.it (M.G.D.C.); mena.ritota@crea.gov.it (M.R.)

**Keywords:** zinc, PDO and traditional cheeses, nutritional evaluation, dietary reference intake

## Abstract

Zinc is an essential mineral which plays a key role in several important biological processes in the human body. The determination of its level in food matrices can contribute to the food quality characterization and to the adequacy of the diet. Animal food products generally have a higher zinc content compared to vegetables. Among them, dairy products consumption can provide a great contribution to the zinc reference intakes. In this study, different Italian cheeses (38 Protected Denomination of Origin and 9 Traditional) were evaluated for their zinc content. Cow cheeses generally showed the highest zinc content (1.83–7.75 mg/100 g cheese), followed by sheep cheeses (1.34–3.69 mg/100 g), and cheeses from mixed milk (0.39–4.54 mg/100 g). The only cheese from buffalo milk (Mozzarella di Bufala Campana PDO) showed a zinc content of 2.14 mg/100 g. The great variability in the zinc content observed among the samples is the result of the influence of several factors, such as the feeding system, the species (cow, sheep, goat, and buffalo), and the cheese-making. Most of the samples resulted in a great contribution (>10%) to the zinc Daily Reference Intake set by EU (10 mg/day), with only two samples contributing to less than 4%.

## 1. Introduction

Zinc, a stable divalent cation, is one of the most abundant elements in the world. In the human body, zinc is the second micronutrient after iron, and it plays a key role in several important biological processes [1]. According to numerous authors [1,2,3,4,5], three different functions can be ascribed to zinc: catalytic, structural, and regulatory roles. Many enzymes require zinc as catalyst, such as oxidoreductases, transferases, hydrolases, lysases, isomerases, and ligases [6], where zinc may activate these enzymes by serving as an electron acceptor. The zinc structural role is related to its ability to promote the folding of proteins into three-dimensional configurations, involving above all cysteine and histidine [2], and because it is considered of critical importance in maintaining the structure of metalloproteins [4]. Finally, it has recently been shown that zinc plays an essential role in polynucleotide transcription and translation, and thus in the processes of genetic expression (such as expression of the metallothionein gene, apoptosis, and synaptic signaling) [5].

As zinc is involved in several functions, its content in the human body ranges from 1.5 to 2.6 g in adults, with bone and skeletal muscle accounting for the highest amount (about 86%), followed by skin (4.2%), and liver (3.4%) [3]. However, zinc is present in all organs, tissues, fluids, and secretions of the body. There is no storage compartment for zinc in the body, and the zinc losses (through feces, urine, sweat, menstrual flow, loss of hair, nails, and desquamated skin) must be replaced by dietary intake. The maintenance of zinc homeostasis is essential for survival, hence, regulations in zinc absorption and excretion are useful to maintain zinc homeostasis [7].

If the supply of dietary zinc is insufficient, biochemical abnormalities and clinical signs may develop, including pregnancy outcome, physical growth, susceptibility to infection, and neurobehavioral development [2,6,8]. This is why infants, children, pregnant, and lactating women are at greater risk of zinc depletion compared to adults.

For this reason, great efforts have been done to estimate the global prevalence of zinc deficiency using accurate and easily measurable indicators of zinc status. A working group made up by the World Health Organization (WHO), the United Nations International Children’s Emergency Fund (UNICEF), the International Atomic Energy Agency (IAEA), and the International Zinc Nutrition Consultative Group (IZiNCG) recommend the blood plasma or the serum zinc concentration as biomarker of the zinc deficiency risk in populations [9].

Many food items are a valuable source of zinc [2]: animal foods, such as liver, meat, seafood, eggs, and dairy products, have higher zinc contents compared to vegetables (beans, cereal, tubers, vegetables, and fruits). However, meal studies based on the investigations of the real utilization of dietary zinc showed the presence of different factors affecting zinc absorption [10,11]. Among them, the ability of myo-inositol hexaphosphate (also known as phytic acid) to interfere with the zinc absorption is the most studied [4,12,13,14,15,16,17]. Phytic acid chelates cations, forming insoluble complexes with minerals that, in this way, cannot be digested or absorbed by humans [18]. However, among the phosphorylated forms of inositol phosphate, the hexaphosphate and pentaphosphate forms inhibited zinc absorption, while the tetraphosphate and triphosphate forms had no significant effect [16]. It is worth highlighting that dietary proteins can modify the effects of phytate [14], since animal proteins have been shown to improve zinc absorption from a phytate-containing diet [19].

Many authors suggest [6,16] the phytates/zinc molar ratio in meals or diets as a useful indicator of the phytates degree in depressing zinc absorption. In a meal with a molar ratio above 15, for example, the zinc absorption is typically less than 15% [19].

Plant-based diets, containing high phytate amounts, provide less available zinc than meat-based diets [15], while animal source foods, such as dairy products, do not contain phytate and therefore have a phytate/zinc molar ratio equivalent to zero. In this regard, some authors [11] showed that zinc bioavailability in soy-based beverage was much lower (≈20%) compared to bovine milk, where the zinc bioaccessible fractions ranged from 59% to 83%, just because of the presence of phytic acid in the soy-based beverage samples. The authors [11] also observed no significant differences among the samples of whole, semi-skimmed and skimmed milk, showing that the lipid content does not seem to affect the zinc bioaccessibility in bovine milk.

The role of calcium in affecting zinc availability is controversial: it does not seem to have any direct effect on zinc absorption [20] but, in the presence of phytate, calcium may increase the phytate inhibitory capacity. Negative effects of some ions, such as iron, on zinc absorption have been reported, while soluble organic substances of low molecular mass, such as amino acids or citric acid, facilitate zinc absorption [3]. Additionally, caseinophosphopeptides (CPPs) seem to increase zinc absorption [21,22]. They can be released by enzymatic digestion of dairy products or during cheese-making [23]. CPPs contain three serine phosphate clusters followed by two glutamic acid residues, and they have a strong capacity to fix divalent cations, such as calcium, iron or zinc, by forming soluble organophosphate salts [21,22].

Hence, since zinc is an essential trace element with a great nutritional importance, the determination of its level in food matrices can contribute to the characterization of the food quality and to the adequacy of the diet. However, it can also result in being toxic for humans when taken in excess [24,25], so much so that the Provisional Maximum Tolerable Daily Intake (PMTDI) of zinc has been fixed to 1.00 mg/kg body weight [26]. Therefore, its determination in food products is of fundamental importance.

In this context, the aim of this work is to update food composition databases with reliable and representative data, with a focus on the zinc content in Protected Denomination of Origin (PDO) and Traditional Italian cheeses. PDO products have the strongest links to the place in which they are made, and every part of the production, processing and preparation process must take place in the specific region. Traditional cheeses are, instead, typical and local products whose processing, preservation, and seasoning methods have been consolidated over time almost since 25 years according to traditional Italian Regulation [27]. Since PDO and Traditional Italian cheeses are food items largely consumed in the European Union (EU), this study also aims to provide the nutritional adequacy of zinc in these cheeses, according to the UE regulation and European Food Safety Authority (EFSA) Population Reference Intakes for zinc [13,28].

## 2. Results and Discussion

### 2.1. Zinc Levels in PDO and Traditional Italian Cheeses

Table 1 shows the zinc content of PDO and Traditional cow cheese samples (expressed in wet weight): the mean data range from 2.28 mg/100 g (Taleggio) and 7.75 mg/100 g (Piave Vecchio) in PDO cheeses, while among Traditional cheeses Giuncata shows the lowest zinc amount (1.83 mg/100 g) and Provola delle Madonie (18 months ripening) has the highest zinc content (7.18 mg/100 g). The differences in these cheese samples partially reflect their water contents [29].

These data are quite similar to those reported by Gambelli et al. [30], especially for the zinc content of Gorgonzola, Caciocavallo, Provolone, and Grana. Kedzierska-Matysek et al. [31] reported similar values for Grana Padano and Fontina zinc contents, 5.00 and 3.49 mg/100 g, respectively. Bontempo et al. [32], in the evaluation of the mineral contents of several Italian cheeses, reported comparable Asiago zinc value (6.50 mg/100 g on a dry weight), while the zinc levels of Fontina, Montasio, Puzzone, Spressa, and Toma were slightly lower than those obtained for the same cheeses in this study. Additionally, Manuelian et al. [33] reported lower zinc levels for Asiago, Grana Padano, Montasio, Parmigiano Reggiano, Piave, Fontina, Provolone, Gorgonzola, and Taleggio, compared to the results obtained in this work, and the data reported by some authors [34,35] for the zinc content of Quartirolo cheese were lower than that obtained in this study.

The zinc content of Parmigiano Reggiano (>18 months ripening) is in the same order of magnitude of the data reported by Kedzierska-Matysek et al. [31], where the zinc values varied between 4.25 and 4.53 mg/100 g. The zinc amount in Giuncata vaccina abruzzese, a soft dairy product for immediate consumption, is comparable with the results obtained by Ianni et al. [36], who reported a zinc value of 2.07 and 1.99 mg/100 g for the same cheese, while the zinc level reported by Barone et al. [37] was lower (0.40 mg/100 g). The zinc contents of Caciocavallo reported by Ianni et al. [38], ranging from 4.36 to 4.20 mg/100 g, are in the same order of magnitude of the data reported for the same cheese in this study and by Gambelli et al. [30].

Table 1 also shows the same type of cheeses produced with milk where cows were bred in indoor or grazed the alpine pasture: this is the case of Nostrano Valtrompia (PDO cheese), Bagoss and Formaggella della Val Trompia (Traditional cheeses). The zinc content decreases (*p* < 0.05) in the cheese samples made with milk from indoor feeding only in Formaggella della Val Trompia cheeses. These results are confirmed even when performing statistical analyses on a dry basis (data not shown). The influence of the feeding system, as well as the season, on milk composition is already known [39]. However, finding a univocal relation between the feeding system and the zinc content in cheese is very difficult due to the influence of several factors: (i) there is a great variability in the mineral contents of mountain pasture plants [40]; (ii) the intensity of grazing could affect mineral levels of the forage [41]; zinc values and its threshold values in soils are not easy to evaluate due to several variables involved [42]; and zinc content in milk could be affected by zinc supplementation in the feeding system [43,44], even if zinc levels in cheese depend on cheese-making processes [45,46,47,48], with curdling and salting being the two phases mainly influencing cheese mineral composition [34]. In confirmation of all these variables simultaneously affecting the zinc content of cheese, there are the studies of Coni et al. [34] and Lucas et al. [49], according to which no influence of the milk season and feeding practices, respectively, on the zinc content of cow cheeses was observed.

Among Traditional Italian cheeses made from cow milk, there seems to be a ripening influence on the zinc content: this is the case of Provola delle Madonie, a pasta filata cheese, in which an increasing trend of the zinc level from unripened to 16 months ripening is observed. Actually, since the same cheeses come from the same farm, the differences are only due to the decreasing water content with ripening, so much so that the significance differences disappear when performing statistical analysis on a dry basis (data not shown). Table 2 shows the zinc content in PDO and Traditional sheep cheeses (expressed in wet weight). According to some authors sheep cheeses are a good source of zinc [50]. In PDO sheep samples, the mean values of zinc range from 2.72 mg/100 g (Formaggio di Fossa di Sogliano) to 3.69 mg/100 g (Canestrato Pugliese), while in Traditional sheep cheeses the zinc values vary from 1.34 (Giuncatella abruzzese) to 3.56 mg/100 g (Pecorino d’Abruzzo, 6 months ripening). In PDO sheep samples, the mean values of zinc range from 2.72 mg/100 g (Formaggio di Fossa di Sogliano) to 3.69 mg/100 g (Canestrato Pugliese), while in Traditional sheep cheeses the zinc values vary from 1.34 (Giuncatella abruzzese) to 3.56 mg/100 g (Pecorino d’Abruzzo, 6 months ripening).

Pecorino d’Abruzzo, a largely consumed cheese in Italy, shows on average 2.98 mg/100 g of zinc, and this value is comparable with the results obtained by Gambelli et al. [30] who reported a mean value of 3.37 mg/100 g for Pecorino-type cheese. Kedzierska-Matysek et al. [31], instead, reported a lower zinc content in Pecorino Toscano compared to the same cheese analysed in this study (1.77 vs. 2.81 mg/100 g).

Table 3 reports the zinc content in PDO cheeses from buffalo and mixed milk (expressed in wet weight). Mozzarella di Bufala Campana PDO shows 2.14 mg/100 g of zinc, with a similar value reported by Gambelli et al. [30] and Bontempo et al. [51] for the same cheese, while Manuelian et al. [33] observed a lower zinc content in Mozzarella di Bufala Campana PDO (1.34 mg/100 g).

In cheeses from mixed milk, instead, the zinc value ranges from 2.07 to 4.54 mg/100 g (Murazzano and Bra duro, hard type, respectively), excluding Robiola di Roccaverano and Ricotta Romana PDO, where the zinc values are respectively 0.39 and 0.36 mg/100 g.

Ricotta Romana PDO is not really a cheese, but a dairy product obtained from whey (sheep whey about 85%). As the soluble fraction of sheep milk the zinc amount is 8.4% [52], the level of this element observed in Ricotta Romana is quite low (0.36 mg/100 g). For the same reason, the data reported by Coni et al. [53] revealed that the zinc content in Ricotta-type cheese was very low (0.16 mg/100 g). The result obtained in this study is also comparable with the data of Gambelli et al. [30], where the zinc content in Ricotta-type cheese (from cow and sheep milk) is 0.35 mg/100 g.

**Figure 1:**
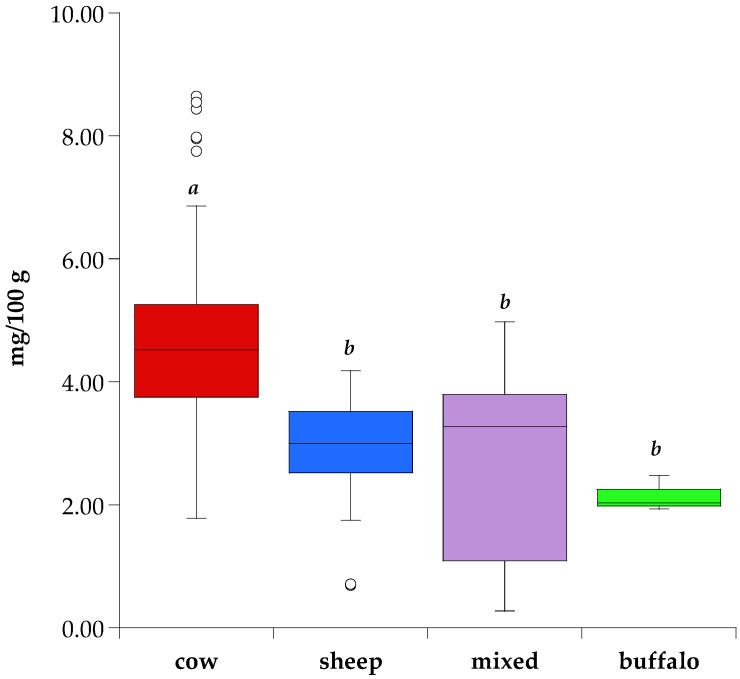
Box plots of the zinc level in cow, sheep, mixed, and buffalo milk cheeses. Significant differences (*p* < 0.05) are identified with different letters (*a*, *b*).

In this study cow milk cheeses result in higher (*p* < 0.05) average zinc content (4.61 mg/100 g) compared to sheep, mixed, and buffalo cheeses (3.00, 2.60, and 2.14 mg/100 g, respectively) as reported in Figure 1. The high variability in mixed cheeses is due to the great heterogeneity of the samples, made with different technological processes and with different percentages of milk from various animal species.

Additionally, Manuelian et al. [33] evaluated the zinc content in cheeses with milk from different species reporting the following trend: cow > sheep > buffalo >> goat. Similarly, Martin-Hernandez et al. [54] observed a lower zinc content in goat cheeses than cow and sheep cheeses. Since the differences in the mineral composition of milk from different animal species and breeds are very small [47,54], differences in the mineral composition of cheese are mainly due to technological processes.

In the case of sheep cheeses, a high zinc content would be expected due to the high zinc amount of sheep milk. According to Raynal-Ljutovac et al. [55], in fact, sheep milk is richer in zinc than cow milk and goat milk, but these results are not often confirmed by other literature studies [56]. However, the sheep cheeses analysed in this study, most of which are Pecorino-type cheeses, show low zinc levels (both on a wet and dry basis). This could be due to the typical technological process of Pecorino-type cheese: in this cheesemaking after coagulation, the curd is broken into the size of a rice grain. Breaking the curd into very small granules could promote the consequent release of zinc into the whey. Furthermore, the cheese salting, which in the case of Pecorino is of greater extent compared to other cheeses [32,33,57,58,59], has been revealed to cause a decrease in the content of some elements, including zinc, after curdling [45]. Even in the case of Fiore Sardo PDO [60], another sheep cheese resulted with a low zinc content, the breaking of the curd is very strong and lasts for about 3 min, so as to reduce it into granules similar to a millet grain.

Concerning cheeses from goat milk, only one type of this sample was available for this study, namely Robiola di Roccaverano. Besides the fact that goat milk does not have a high zinc content, the lowest zinc amount observed in this cheese certainly reflects its manufacturing process (acid coagulation), which involves an acid coagulation before adding the rennet, resulting in a cheese characterized by high moisture content and a fragile curd [61]. Because of the acidification, the stability of casein micelles decreases, their charge becomes lower [62] with the loss of calcium and other minerals, such as zinc, in the whey. This is due to the tendency of zinc to form stable complexes with peptides and proteins [63] and it has been shown that even more than 90% of the zinc in sheep milk and about 87% of the zinc in goat milk is present in the micellar fraction, mainly bound to caseins [52], with similar distribution reported for cow milk [63]. The low zinc content in Robiola di Roccaverano is comparable with the levels detected in other cheeses with acid coagulation technology such as cottage cheese: actually, some authors founded 0.48 mg/100 g of zinc in cottage cheese [30]. Similarly, Moreno-Rojas et al. [64] reported a zinc value of 0.54 mg/100 g for Los Balanchares, a cheese produced from goat milk by acid coagulation, compared to other Spanish cheeses produced through enzymatic coagulation, the zinc levels of which ranged between 1.71 and 3.84 mg/100 g. Similarly, Lucas et al. [65] observed a very low zinc content in a goat cheese from lactic coagulation (Rocamadour) compared to other cow cheeses from rennet coagulation.

Wide variations have been reported in the zinc levels of buffalo milk [66], but data reported in the literature show that it contains moderate zinc amounts (0.57 mg/100 g [67]; 0.44 mg/100 g [68]; 0.41 mg/100 g [69]; 0.63 mg/100 g [70]) compared to milk from other species. In this study, one cheese from pure buffalo milk was evaluated, i.e., Mozzarella di Bufala Campana PDO whose values are comparable with sheep and mixed cheeses (Figure 1). According to its product specification [71], the acidification of milk and curd are obtained by adding natural whey; then maturation of the curd takes place under whey over a variable time period (maximum 5 h), during which pH gradually decreases, contributing to modify the structure and rheological properties of the curd, and promoting cheese spinning. The pH decrease of milk and curd in Mozzarella leads to a decrease in mineral content [33], therefore a not very high zinc content would be expected. This is what is observed in Mozzarella di Bufala Campana PDO on wet weight but, performing analysis on a dry weight surprisingly reveals a higher zinc content compared to sheep cheeses, as well as to some cow cheeses (Taleggio and Castelmagno). Probably the starting zinc value of the buffalo milk was very high, and the curd maturation into the whey could promote the reabsorption of the zinc lost through the whey.

### 2.2. Nutritional Evaluation of Zinc in PDO and Traditional Italian Cheeses

Dairy products are food items largely consumed: in Europe the average daily consumption of cheese is about 35.3 mg/day in adults [72], with Italy being the country with the highest cheese consumption. According to the Italian National Food Consumption Survey 2005–2006 [73], in fact, the Italian daily intake of cheese is 59.6 g for adults (18–64.9 years), with “Milk, milk products and substitutes” category providing 21% of the zinc daily intake [74].

According to the Daily Reference Intake (DRI) set by the European Parliament and the Council of the European Union [28], the requirement for zinc in adults is estimated at 10 mg/day. In order to calculate the contribute of cheese consumption to the zinc DRI, it is necessary to establish the amount of the cheese servings; however, the daily serving sizes of cheeses are not well defined all over the world, and they may vary in different countries. A recent Italian position paper [75] have defined two different servings for cheeses based on the fat amount: a serving size of 50 g if the fat content of cheese is more than 25 g/100 g edible weight, and a serving size of 100 g if the fat content is less than 25 g/100 g edible weight.

Table 4, Table 5 and Table 6 report the zinc Estimated Dietary Intakes (%) for one serving (50 or 100 g) of each cheese. Based on the European DRI [28] the percentage of the zinc intake ranges from 11.4 (Taleggio) to 38.7% (Piave Vecchio) with 50 g of cow cheeses, reaching 48.7% in the case of 100 g serving of unripened Provola delle Madonie (Table 4). Concerning cheeses from sheep milk, the percentage of the zinc intake is between 10.9% (Pecorino d’Abruzzo 7 months ripening) and 18.5% (Canestrato Pugliese) (Table 5), while in cheeses from mixed milk the data range from 1.9% (Robiola di Roccaverano) to 22.7% (Bra hard type) (Table 6). With 100 g of Ricotta Romana the percentage of the zinc intake is only 3.6%, while with a serving (100 g) of Mozzarella di Bufala Campana PDO this intake is 21.4%.

These results show that the Estimated Dietary Intake calculated for zinc in the cheese samples provides a significant contribution to the DRI set by EU [28] in most of the samples analyzed, corroborating the statement that cheeses are a good source of this essential element. Indeed, among 57 cheeses evaluated, two samples (Robiola di Roccaverano and Ricotta Romana) contribute to less than 4% of the Daily Reference Intake for zinc, 27 samples provide 10–20% of the zinc DRI, 23 cheeses ensure an average percentage of zinc intake ranging from 20 to 30%, while 5 samples even exceed 30%, with Provola delle Madonie reaching the highest value (almost 50%). These results are much higher than those reported by Gambelli et al. [30], who observed a zinc daily intake by Italian cheese consumption of about 10%. Similarly, Barone et al. [37] estimated a zinc intake ranging from 3.09% to 6.50% through consumption of some Traditional Italian cheeses. The authors [37], however, referred to a general cheese serving of 65 g/person/day and to the Recommended Dietary Allowances (RDAs) for zinc set by the Food and Nutrition Board of the Institute of Medicine [76], that are 11 mg/day for men and 8 mg/day for women. Additionally, Bilandžić et al. [77] referred to the same RDAs in evaluating the contribution of cheeses from Croatia to the zinc RDA: the authors observed very high values of EDI (%), with data ranging from 4.9% (in male through fresh cheese consumption) to 22.8% (in female through hard fat cheese consumption). However, the authors [77] referred to a cheese serving of 200 g/day per adult, so their EDIs values can be considered much lower than those reported in this work.

It must be highlighted, however, that the Daily Reference Intake for zinc set by EU [28] has some limitations: it does not consider that worldwide Zn recommendations are greater in men than in women because of the differences in protein requirement, and it does not consider the difference in diet composition and in particular the inhibitory effect of dietary fibre, phytate, or polyphenols [78] on the zinc bioavailability and absorption. Among these parameters, phytic acid is one of the most important because it chelates cations, forming insoluble complexes that cannot be digested or absorbed by humans, thus leading to a reduction in the zinc absorption. There is a wide variation in phytate intake among diets or countries: a mixed diet can contribute for about 300 to 800 mg/day of phytate, up to 1400 mg/day for diets with a high proportion of unrefined cereal grain products and legumes, while in vegetarian diets the phytate intake could reach 1600–2500 mg/day [13].

For this reason, based on the available data from zinc absorption studies and on the molar ratios phytate/zinc, WHO/FAO [14] has classified diets into three categories according to the potential bioavailability of their zinc, namely high, moderate, and low zinc availability. Consequently, three different Recommended Nutrient Intakes (RNIs) for dietary zinc (mg/day) in adults (males 19–65 years 65 kg body weight, and females 19–65 years 55 kg body weight) were set according to the three diets differing in zinc bioavailability.

However, more recently, EFSA panel has suggested different zinc Population Reference Intakes (PRIs) for adult (18–79 years) men (79.4 kg body weight) and women (68.1 kg body weight) based on four phytate intake levels (300, 600, 900, and 1200 mg/day); these PRIs range from 7.5 to 12.7 mg/day of zinc for women, and from 9.4 to 16.3 mg/day for men [13].

According to these PRIs set by EFSA, Table 4, Table 5 and Table 6 also report the % Estimated Dietary Intakes (EDIs) for zinc in cheeses based on the four different phytate intake levels. Comparing the EDIs calculated according to the EU regulation [28] or according to the EFSA recommendation [13], it is worth observing that the zinc dietary intakes for a 600 mg/day phytate content are quite similar to the EFSA recommendations, ranging, in all studied cheeses, from 1.7 to 41.6% in male and from 2.1 to 52.3% in female.

It is well known that there are discrepancies among the worldwide recommended nutrient values: as an example, low-income countries follow the WHO recommendations, while the USA and Canada set different recommendations [79,80], and Australia and New Zealand follow their own recommendations [81]. These differences are probably due to different lifestyles and/or types of diet. The new levels proposed by EFSA could be a problem, for example, in a low-income country, where there is a high consume of unrefined cereal, while they could be easier to apply in European countries.

Since there are a lot of public health implications due to zinc deficiency, IZiNCG [2] suggested three intervention strategies: supplementation, fortification, and dietary diversification/modification. However, it is worth highlighting that, because of the coexistence of several micronutrient deficiencies, all the intervention programs should be linked each other in order to obtain the maximum effect and an efficient use of resources [2].

As reported by Freeland-Graves et al. [78], an effort could be made to harmonize dietary standards, and the enhancement of micronutrient bioavailability could be improved by the adoption of food preparation or food processing techniques.

Finally, the Estimated Daily Intake calculated in adults for each cheese was also compared to toxicological values, which in the case of zinc is the Provisional Maximum Tolerable Daily Intake (PMTDI) set by FAO/WHO [26]: all cheese samples were lower than the above mentioned PMTDI (1.00 mg/kg body weight), suggesting that, in terms of food security, the consumption of the evaluated cheeses does not cause any health hazards to consumer.

## 3. Materials and Methods

### 3.1. Samples

A total of 57 PDO and Traditional Italian cheeses were purchased from different local groceries, supermarkets, or local farms in Italy. In particular, this study evaluated cheese samples from milk of different animal species, belonging to 47 labels (38 PDO and 9 Traditional), and classified as below:

- 23 PDO and 7 Traditional cow milk cheeses
- 6 PDO and 2 Traditional sheep milk cheeses
- 1 Mozzarella PDO cheese made with buffalo milk
- 7 PDO cheeses made with milk of different animal species (various percentages of cow, sheep, and goat milk according to their production specifications)
- 1 PDO Ricotta a dairy product made with a mixture of sheep whey and milk.

For each cheese three different wheels were collected.

All dairy products were sampled and grated according to the reference method [82] and analysed in triplicate.

### 3.2. Chemicals

Ultrapure water was prepared by an ion exchange system to >18 mΩ resistivity (ElgaPurelab Ultra, Veolia, UK). All reagents were of the highest available purity level and the zinc standard was obtained from Merck (Darmstadt, Germany).

### 3.3. Zinc Analysis and Equipment

Zinc was determined according to a modification of the AOAC method [83]. The cheese samples were analysed for their zinc content after ashing: 1 g of sample were weighed into platinum crucibles, dried 1 h in 100 °C forced air oven and ashed into the furnace at 525 °C for 16 h; ashes were dissolved in 1 mL HNO_3_ 65% and then filled to a final volume of 50 mL with HNO_3_ 1% (*v*/*v*).

Zinc was determined using an Atomic Absorption Spectrometer AAnalyst 300 (Perkin-Elmer, Norwalk, CT, USA). A standard stock solution of zinc was used (1000 mg/L, zinc standard solution traceable to SRM from NIST). Individual working standard solutions were prepared at three different levels by dilution (0.25, 0.50, and 1.00 µg/mL) in HNO_3_ 1% *v/v.* The study involved a Standard Reference Material (SRM 1846 Infant formula) certified for zinc concentration.

### 3.4. Estimated Dietary Intake

The Estimated Dietary Intake (EDI) was considered as the percentage contribution of the zinc intake through each cheese consumption to the daily reference intakes set by different international authorities [28,72] for adults. It was calculated using the following Equation (1):

EDI (%) = (C × IR) × 100/DRI (or PRI)(1)

where C represents the zinc concentration in cheese (mean value expressed as mg/g), IR is the ingestion rate (g/day) of cheese, equivalent to a serving of 50 g/person/day or 100 g/person/day [75], DRI is the Daily Reference Intake set by the European Parliament and the Council of the European Union for zinc in adults [28], and PRI is the Population Reference Intakes set by the European Food Safety Authority (EFSA) for zinc in men and women at different phytate levels [13].

### 3.5. Statistical Analysis

One-way analysis of variance (ANOVA) test, coupled with the Tukey’s post hoc test, was performed to evaluate significant differences (*p* < 0.05) among the mean values. Statistical analysis was performed using XL-STAT Base 18.06 (Addinsoft 1995–2017).

## 4. Conclusions

Even if in trace concentrations, zinc represents an essential element for the human body, but consumption in levels exceeding recommended concentrations could result in adverse consequences. Therefore, its intake through the diet must be carefully monitored and at the same time guaranteed. The analysis of different Italian cheeses (both PDO and Traditional) evaluated in this study showed a great variability in their zinc content, which values ranged from 0.36 to 7.75 mg/100 g cheese. Further studies will be carried out in order to confirm the obtained results. Differences in the zinc levels of cheeses may be ascribed to a several number of factors: milk origin (cow, sheep, goat, and buffalo), animal feeds quality, and characteristics of the milk, which are in turn influenced by different variables (i.e., breed, physiological condition of the animal, stage of lactation, etc.). Cheese-making processes are indeed the mainly responsible for the observed differences in the zinc content of the various cheeses, but contamination phenomena (from the process equipment, packaging, and/or storage) could also play an important role.

The cheese analysed in this work provided a great contribution to the zinc reference intakes: most of the samples (about 50%) provide more than 20% of the European Daily Reference Intake for zinc (10 mg/day) with one serving of cheese. Furthermore, the consumption of the cheese evaluated in the study do not represent a potential public health risk, since no cheeses serving exceeded the Provisional Maximum Tolerable Daily Intake set for zinc (1.00 mg/kg body weight). Therefore, the consumption of cheese as a valuable source of zinc was once again confirmed.

## Figures and Tables

**Table 1:** Zinc content in PDO and Traditional Italian cow cheeses (mg/100 g).

Samples	Samples	* N	Min	Max	Mean	Std Dev
PDO	Asiago	5	4.22	5.25	4.61	0.44
PDO	Asiago Pressato	10	3.47	5.39	4.08	0.66
PDO	Bitto	2	4.06	4.90	4.48	0.59
PDO	Caciocavallo Silano	2	4.38	4.59	4.48	0.15
PDO	Casera	2	4.81	5.10	4.93	0.15
PDO	Fontina	6	3.49	4.69	3.95	0.50
PDO	Formai de Mut	2	4.59	4.84	4.72	0.18
PDO	Gorgonzola	8	2.43	3.35	2.94	0.29
PDO	Grana Padano (>16 months)	5	5.33	5.76	5.48	0.18
PDO	Montasio	4	4.16	5.05	4.63	0.39
PDO	Monteveronese	2	3.77	4.36	4.07	0.42
PDO	Monteveronese d’allevo	3	4.02	4.35	4.29	0.25
PDO	Nostrano Valtrompia					
	*Alpine pasture*	4	3.94	5.27	4.65	0.71
	*indoor*	4	4.95	5.06	5.01	0.05
PDO	Parmigiano Reggiano (>18 months)	5	5.01	5.54	5.29	0.19
PDO	Piave Vecchio	2	7.53	7.96	7.75	0.31
PDO	Provolone Valpadana	5	3.67	4.51	4.17	0.31
PDO	Puzzone di Moena	2	4.07	4.35	4.21	0.20
PDO	Quartirolo Lombardo	2	2.99	3.84	3.42	0.60
PDO	Ragusano	4	4.45	5.52	4.85	0.47
PDO	Spressa delle Giudicarie	2	5.73	5.94	5.83	0.15
PDO	Stelvio	2	3.92	4.19	4.05	0.19
PDO	Taleggio	5	2.04	2.42	2.28	0.15
PDO	Toma Piemontese	5	3.20	4.28	3.95	0.43
Traditional	Bagoss					
	*Alpine pasture*	6	5.25	7.98	6.23	1.36
	*indoor*	4	5.77	6.06	5.90	0.14
Traditional	Caciotta di Raiano	3	3.53	3.58	3.55	0.03
Traditional	Formaggella della Val Trompia					
	*Alpine pasture*	4	4.14	4.72	4.45	0.28
	*indoor*	4	3.18	3.58	3.39	0.17
Traditional	Giuncata abruzzese Vaccina	2	1.78	1.88	1.83	0.07
Traditional	Provola delle Madonie					
	*Unripened*	6	4.52	5.25	4.87	0.37
	*4 months*	6	5.70	6.86	6.26	0.60
	*6 months*	6	5.75	8.65	7.18	1.51
	*Smoked*	6	4.43	5.26	4.79	0.37

* N = number of cheeses obtained from different farmers.

**Table 2:** Zinc content in PDO and Traditional Italian sheep cheeses (mg/100 g).

Samples		* N	Min	Max	Mean	Std Dev
PDO	Canestrato Pugliese	2	3.49	3.89	3.69	0.19
PDO	Fiore Sardo	5	2.20	3.74	3.25	0.61
PDO	Formaggio di Fossa di Sogliano	2	2.67	2.77	2.72	0.07
PDO	Pecorino Romano	6	3.15	4.18	3.66	0.38
PDO	Pecorino Sardo	4	2.62	4.11	3.60	0.67
PDO	Pecorino Toscano	6	2.31	3.54	2.81	0.50
Traditional	Giuncatella abruzzese	5	0.69	1.77	1.34	0.58
Traditional	Pecorino d’Abruzzo					
	*Primo Sale*	8	2.38	3.24	2.89	0.29
	*2 months*	3	3.06	3.14	3.10	0.04
	*3 months*	2	2.82	2.88	2.85	0.04
	*4 months*	10	2.34	3.43	2.79	0.39
	*6 months*	3	3.41	3.70	3.56	0.15
	*7 months*	2	2.08	2.27	2.18	0.14
	*18 months*	2	3.46	3.53	3.50	0.05

* N = number of cheeses obtained from different farmers.

**Table 3:** Zinc content in PDO Italian cheeses from buffalo milk, from mixed milk, and from sheep whey (mg/100 g).

Samples		* N	Min	Max	Mean	Std Dev	Milk Origin
PDO	Mozzarella di Bufala Campana	3	1.93	2.47	2.14	0.29	buffalo
PDO	Bra duro (*hard type*)	2	4.12	4.97	4.54	0.60	cow (small amounts goat or sheep milk)
PDO	Bra Tenero (*soft type*)	3	3.58	3.85	3.69	0.14	cow (small amounts goat or sheep milk)
PDO	Castelmagno	3	1.73	3.35	2.64	0.83	cow (small amounts goat or sheep milk)
PDO	Raschera	2	3.74	4.05	3.90	0.22	cow (small amounts goat or sheep milk)
PDO	Caciotta d’Urbino	2	3.19	3.19	3.19	0.09	sheep (70–80%) + cow milk (30–20%)
PDO	Murazzano	2	1.99	2.13	2.07	0.07	sheep or sheep (>60%) + cow (<40%) milk
PDO	Robiola di Roccaverano	2	0.37	0.41	0.39	0.03	goat or goat (50%) + cow/sheep (50%) milk
PDO	Ricotta Romana	2	0.27	0.45	0.36	0.13	sheep whey + sheep milk (max 15%)

* N = number of cheeses obtained from different farmers.

**Table 4:** Estimated Dietary Intake (%) of zinc in adults calculated according to the reference values of EU [28] and EFSA [13], with a serving (50 g or 100 g) of cheese from cow milk.

			Male				Female			
Cheeses	Serving	Adults	300 mg phytate/day	600 mg phytate/day	900 mg phytate/day	1200 mg phytate/day	300 mg phytate/day	600 mg phytate/day	900 mg phytate/day	1200 mg phytate/day
		° DRI 10 mg/day	^§^ PRI 9.4 mg/day	PRI 11.7 mg/day	PRI 14.0 mg/day	PRI 16.3 mg/day	PRI 7.5 mg/day	PRI 9.3 mg/day	PRI 11.0 mg/day	PRI 12.7 mg/day
PDO	(g)	Estimated Dietary Intake (%)								
Asiago	50	23.0	24.5	19.7	16.4	14.1	30.7	24.8	20.9	18.1
Asiago Pressato	50	20.4	21.7	17.4	14.6	12.5	27.2	21.9	18.5	16.1
Bitto	50	22.4	23.8	19.2	16.0	13.8	29.9	24.1	20.4	17.6
Caciocavallo Silano	50	22.4	23.8	19.1	16.0	13.7	29.9	24.1	20.4	17.6
Casera	50	24.6	26.2	21.1	17.6	15.1	32.8	26.5	22.4	19.4
Fontina	50	19.8	21.0	16.9	14.1	12.1	26.3	21.2	18.0	15.6
Formai de Mut	50	23.6	25.1	20.2	16.8	14.5	31.5	25.4	21.4	18.6
Gorgonzola	50	14.7	15.6	12.5	10.5	9.0	19.6	15.8	13.3	11.6
Grana Padano (>16 m)	50	27.4	29.1	23.4	19.6	16.8	36.5	29.5	24.9	21.6
Montasio	50	23.2	24.6	19.8	16.5	14.2	30.9	24.9	21.1	18.2
Monteveronese	50	20.3	21.6	17.4	14.5	12.5	27.1	21.9	18.5	16.0
Monteveronese d’allevo	50	21.5	22.8	18.3	15.3	13.2	28.6	23.1	19.5	16.9
Nostrano Valtrompia *(Alpine pasture)*	50	23.3	24.8	19.9	16.6	14.3	31.0	25.0	21.2	18.3
Nostrano Valtrompia *(indoor)*	50	25.1	26.7	21.4	17.9	15.4	33.4	26.9	22.8	19.7
Parmigiano Reggiano (>18 m)	50	26.4	28.1	22.6	18.9	16.2	35.2	28.4	24.0	20.8
Piave Vecchio	50	38.7	41.2	33.1	27.7	23.8	51.6	41.7	35.2	30.5
Provolone Valpadana	50	20.9	22.2	17.8	14.9	12.8	27.8	22.4	19.0	16.4
Puzzone Di Moena	50	21.1	22.4	18.0	15.1	12.9	28.1	22.7	19.2	16.6
Quartirolo Lombardo	50	17.1	18.2	14.6	12.2	10.5	22.8	18.4	15.5	13.4
Ragusano	50	24.2	25.8	20.7	17.3	14.9	32.3	26.1	22.0	19.1
Spressa delle Giudicarie	50	29.2	31.0	24.9	20.8	17.9	38.9	31.4	26.5	23.0
Stelvio	50	20.3	21.6	17.3	14.5	12.4	27.0	21.8	18.4	16.0
Taleggio	50	11.4	12.1	9.7	8.1	7.0	15.2	12.2	10.3	9.0
Toma Piemontese	50	19.8	21.0	16.9	14.1	12.1	26.4	21.3	18.0	15.6
**TRADITIONAL**										
Bagoss *(Alpine pasture)*	50	31.1	33.1	26.6	22.2	19.1	41.5	33.5	28.3	24.5
Bagoss *(indoor)*	50	29.5	31.4	25.2	21.1	18.1	39.3	31.7	26.8	23.2
Caciotta di Raiano	50	17.8	18.9	15.2	12.7	10.9	23.7	19.1	16.2	14.0
Formaggella della Val Trompia *(Alpine pasture)*	50	22.3	23.7	19.0	15.9	13.7	29.7	23.9	20.2	17.5
Formaggella della Val Trompia *(indoor)*	50	16.9	18.0	14.5	12.1	10.4	22.6	18.2	15.4	13.3
Giuncata Vaccina abruzzese	100	18.3	19.4	15.6	13.1	11.2	24.4	19.6	16.6	14.4
Provola delle Madonie unripened	100	48.7	51.8	41.6	34.8	29.8	64.9	52.3	44.2	38.3
Provola delle Madonie 4 m	50	31.3	33.3	26.7	22.3	19.2	41.7	33.6	28.4	24.6
Provola delle Madonie 16 m	50	35.9	38.2	30.7	25.6	22.0	47.8	38.6	32.6	28.3
Provola delle Madonie smoked	50	23.9	25.5	20.5	17.1	14.7	31.9	25.7	21.8	18.8

° DRI = Daily Reference Intake set by the EU Regulation [28] for zinc in adults. ^§^ PRI = Population Reference Intakes set by EFSA [13] for zinc in men and women at different phytate levels.

**Table 5:** Estimated Dietary Intake (%) of zinc in adults calculated according to the reference values of EU [28] and EFSA [13], with a serving (50 g or 100 g) of cheese from sheep milk.

			Male				Female			
Cheeses	Serving	Adults	300 mg phytate/day	600 mg phytate/day	900 mg phytate/day	1200 mg phytate/day	300 mg phytate/day	600 mg phytate/day	900 mg phytate/day	1200 mg phytate/day
		° DRI 10 mg/day	^§^ PRI 9.4 mg/day	PRI 11.7 mg/day	PRI 14.0 mg/day	PRI 16.3 mg/day	PRI 7.5 mg/day	PRI 9.3 mg/day	PRI 11.0 mg/day	PRI 12.7 mg/day
PDO	(g)	Estimated Dietary Intake (%)								
Canestrato Pugliese	50	18.5	19.6	15.8	13.2	11.3	24.6	19.8	16.8	14.5
Fiore Sardo	50	16.3	17.3	13.9	11.6	10.0	21.7	17.5	14.8	12.8
Formaggio di Fossa di Sogliano	50	13.6	14.5	11.6	9.7	8.3	18.1	14.6	12.4	10.7
Pecorino Romano	50	18.3	19.4	15.6	13.1	11.2	24.4	19.7	16.6	14.4
Pecorino Sardo	50	18.0	19.2	15.4	12.9	11.1	24.0	19.4	16.4	14.2
Pecorino Toscano	50	14.1	15.0	12.0	10.0	8.6	18.8	15.1	12.8	11.1
**TRADITIONAL**										
Pecorino d’Abruzzo Primo Sale	50	14.5	15.4	12.4	10.3	8.9	19.3	15.5	13.1	11.4
Pecorino d’Abruzzo 2 m	50	15.5	16.5	13.2	11.1	9.5	20.7	16.7	14.1	12.2
Pecorino d’Abruzzo 3 m	50	14.2	15.2	12.2	10.2	8.7	19.0	15.3	13.0	11.2
Pecorino d’Abruzzo 4 m	50	13.9	14.8	11.9	10.0	8.6	18.6	15.0	12.7	11.0
Pecorino d’Abruzzo 6 m	50	17.8	18.9	15.2	12.7	10.9	23.7	19.1	16.2	14.0
Pecorino d’Abruzzo 7 m	50	10.9	11.6	9.3	7.8	6.7	14.5	11.7	9.9	8.6
Pecorino d’Abruzzo 18 m	50	17.5	18.6	14.9	12.5	10.7	23.3	18.8	15.9	13.8
Giuncatella abruzzese	100	13.4	14.2	11.4	9.5	8.2	17.8	14.4	12.1	10.5

° DRI = Daily Reference Intake set by the EU Regulation [28] for zinc in adults. ^§^ PRI = Population Reference Intakes set by EFSA [13] for zinc in men and women at different phytate levels.

**Table 6:** Estimated Dietary Intake (%) of zinc in adults calculated according to the reference values of EU [28] and EFSA [13], with a serving (50 g or 100 g) of cheese from buffalo milk, from mixed milk, and from sheep whey.

			Male				Female			
Cheeses	Serving	Adults	300 mg phytate/day	600 mg phytate/day	900 mg phytate/day	1200 mg phytate/day	300 mg phytate/day	600 mg phytate/day	900 mg phytate/day	1200 mg phytate/day
		° DRI 10 mg/day	^§^ PRI 9.4 mg/day	PRI 11.7 mg/day	PRI 14.0 mg/day	PRI 16.3 mg/day	PRI 7.5 mg/day	PRI 9.3 mg/day	PRI 11.0 mg/day	PRI 12.7 mg/day
PDO	(g)	Estimated Dietary Intake (%)								
Mozzarella di Bufala Campana	100	21.4	22.8	18.3	15.3	13.1	28.6	23.0	19.5	16.9
Murazzano	50	10.3	11.0	8.8	7.4	6.3	13.8	11.1	9.4	8.1
Castelmagno	50	13.2	14.0	11.3	9.4	8.1	17.6	14.2	12.0	10.4
Bra Tenero *(soft type*)	50	18.5	19.7	15.8	13.2	11.3	24.6	19.9	16.8	14.5
Bra Duro (*hard type*)	50	22.7	24.2	19.4	16.2	13.9	30.3	24.4	20.6	17.9
Raschera	50	19.5	20.7	16.6	13.9	11.9	26.0	20.9	17.7	15.3
Caciotta d’Urbino	50	16.0	17.0	13.6	11.4	9.8	21.3	17.2	14.5	12.6
Robiola di Roccaverano	50	1.9	2.1	1.7	1.4	1.2	2.6	2.1	1.8	1.5
Ricotta Romana	100	3.6	3.8	3.1	2.6	2.2	4.8	3.9	3.3	2.8

° DRI = Daily Reference Intake set by the EU Regulation [28] for zinc in adults. ^§^ PRI = Population Reference Intakes set by EFSA [13] for zinc in men and women at different phytate levels.

## Data Availability

The data presented in this study are available on request from the corresponding author.

## Sample Availability

Samples of the compounds are available from the authors.
